# Mitochondrial replenishment as a treatment for Alzheimer’s Disease

**DOI:** 10.1002/alz.086909

**Published:** 2025-01-03

**Authors:** Gherardo Baudo

**Affiliations:** ^1^ Houston Methodist Research Institute, Houston, TX USA

## Abstract

**Background:**

Findings have demonstrated that mitochondrial dysfunction is vital to Alzheimer’s Disease (AD) pathogenesis and progression. This study explored an innovative treatment strategy involving transfer of polymer‐functionalized, healthy mitochondria to AD neurons. We hypothesized that this organelle transplantation approach would restore mitochondrial function and bioenergetics, preventing aberrant neuronal dynamics associated with AD.

**Method:**

Neuronal cells (SH‐SY5Y) were stimulated with amyloid‐β (Aβ_1‐42_). Mitochondria were functionalized with dextran‐triphenylphosphonium (Dex‐TPP/Mt) and administered to neurons. Internalization of Dex‐TPP/Mt was assessed, as was the effect of mitochondrial transfer on Aβ‐treated SH‐SY5Y cell ROS levels, mitochondrial membrane potential, mitochondrial dynamics, ATP levels, and apoptosis.

**Result:**

Dex‐TPP/Mt underwent efficient internalization into Aβ‐treated SH‐SY5Y cells. The treatment reduced intracellular ROS levels and restored mitochondrial membrane potential. Mitochondrial quality control was also re‐established, as mitochondrial fusion and fission processes were balanced and mitophagy reduced. Dex‐TPP/Mt treated neurons also increased ATP levels. Notably, mitochondrial transfer modulated the Bax/Bcl‐2 ratio, inhibited caspase‐3 activation, and suppressed the pJNK pathway, highlighting reduced neuronal apoptosis that was confirmed by Annexin V assay.

**Conclusion:**

Mitochondrial transfer has potential as a therapeutic approach for AD. The treatment highlighted herein effectively mitigated the impact of Aβ on neuronal health by targeting mitochondrial dysfunction and oxidative stress. This opens several avenues for similar strategies aimed at addressing dysregulated bioenergetics and mitochondrial dynamics in AD.

